# Surgical Smoke—Hazard Perceptions and Protective Measures in German Operating Rooms

**DOI:** 10.3390/ijerph17020515

**Published:** 2020-01-14

**Authors:** Martina Michaelis, Felix Martin Hofmann, Albert Nienhaus, Udo Eickmann

**Affiliations:** 1 Research Centre for Occupational and Social Medicine (FFAS), 79098 Freiburg, Germany; michaelis@ffas.de (M.M.); mahofmann@ffas.de (F.M.H.); 2 Department of Occupational Medicine, Hazardous Substances and Health Sciences, German Statutory Accident Insurance and Prevention in the Health and Welfare Services (BGW), 20357 Hamburg, Germany; 3 Competence Center for Epidemiology and Health Services Research for Dermatology and Nursing (IVDP), University Medical Center, 20251 Hamburg-Eppendorf, Germany; 4 Department of Occupational Medicine, Hazardous Substances and Health Sciences, German Statutory Accident Insurance and Prevention in the Health and Welfare Services (BGW), Hazardous Substances and Toxicology Division, 50968 Cologne, Germany; udo.eickmann@bgw-online.de

**Keywords:** surgical smoke, hazard perceptions, protective measures, survey, surgeons, technical assistant staff

## Abstract

(1) Background: Hazardous substances in surgical smoke that is generated during laser or electrosurgery pose a potential health hazard. In Germany, the Technical Rules for Hazardous Substances (TRGS 525) have included recommendations for appropriate protective measures since 2014. Up to now, no empirical data has been available on the extent to which recommendations have been implemented in practice. (2) Methods: In 2018, 7089 surgeons in hospitals and outpatient practices were invited by email to participate in an online survey. In addition, 219 technical assistants were interviewed. The questionnaire dealt with knowledge of, and attitudes toward, the hazard potential of surgical smoke, as well as the availability and actual use of protective measures. Furthermore, manufacturers and distributors of smoke extraction devices were asked to give their assessment of the development of prevention in recent years. (3) Results: The survey response rate was 5% (surgeons) and 65% (technical assistant staff). Half of all surgeons assumed that there were high health hazards of surgical smoke without taking protective measures. Operating room nurses were more often concerned (88%). Only a few felt properly informed about the topic. The TRGS recommendations had been read by a minority of the respondents. In total, 52% of hospital respondents and 65% of the respondents in outpatient facilities reported any type of special suction system to capture surgical smoke. One-fifth of respondents from hospitals reported that technical measures had improved since the introduction of the TRGS 525. Fifty-one percent of the surgeons in hospitals and 70% of the surgeons in outpatient facilities “mostly” or “always” paid attention to avoiding surgical smoke. The most important reason for non-compliance with recommendations was a lack of problem awareness or thoughtlessness. Twelve industrial interviewees who assessed the situation and the development of prevention in practice largely confirmed the prevention gaps observed; only slight developments were observed in recent years. (4) Conclusions: The low response rate among surgeons and the survey results both indicate a major lack of interest and knowledge. Among other measures, team interventions with advanced training are needed in the future.

## 1. Introduction

### 1.1. Origin and Properties of Surgical Smoke

Laser and electrosurgery produce surgical smoke during the separation, scabbing, and stopping of bleeding through the use of ultrasound or heat [1]. The application of these treatment methods not only causes a considerable odor nuisance, but also releases a mixture of gaseous, vaporous, liquid, and solid substances. These substances include carbon monoxide, acrylonitrile, hydrogen cyanide (hydrocyanic acid), and formaldehyde [2,3,4]. Hill et al. [5] concluded, in an exposure study, that on average the smoke produced daily was equivalent to about 30 cigarettes.

The chemical composition of the substances and the size of the particles emitted vary depending on the process used and the tissue treated [1,6,7]. For example, a peritonectomy releases significant amounts of fine particles [8], while coarser particles are typically produced during laser treatments [3]. It was already shown, in the 1970s, that laser treatments release surgical smoke which also contains active biological components such as cells or cell parts [9]. Infectious agents contained in surgical smoke such as staphylococci or human papillomavirus can cause infections in exposed individuals [2,10,11].

### 1.2. Exposure of Operating Room Staff to Health Hazards Caused by Surgical Smoke

Essential control variables for the exposure of surgeons and operating room nurses are the performance and type of ventilation systems, the type of treatment, the surgical instruments used, the duration of exposure, and the qualifications of the practitioner [1]. Surgical smoke is associated with dose-dependent health complaints such as headache, weakness, nausea, rhinitis, or a burning sensation in the nasopharynx [4,12], but also with more serious conditions such as asthma or pneumonia [13]. A causal relationship between neurotoxic, carcinogenic, and mutagenic substances in surgical smoke [6,11,12,14,15] and tumors appears to be possible and is discussed in case studies [16,17]. Meaningful empirical evidence of causal relationships is not provided by the current study situation, despite the toxic substances contained therein [18,19,20,21].

### 1.3. Protective Measures and Recommendations

It has long been known that surgical masks do not provide adequate protection during laser and electrosurgery due to the small size of the particles [13,22,23,24]. Fit-tested high-filtration particle masks covering the mouth and nose offer better protection but are not considered to be a sufficient substitute for smoke evacuation devices [12,25].

Suction devices are equipped with a special filter system (activated carbon or ultra-low penetration air filter) that enables effective retention of the substances contained in surgical smoke [14]. The systems are usually offered by manufacturers as part of complete packages which contain the suction unit itself and a suction tube or hose. This can be connected either to a surgical handle or to a suction canister. The use of a system where the suction hose can be connected directly to a surgical handle is recommended because it allows direct extraction of smoke at its source, Eickmann 2011 ASU [1]. Despite the great potential of technical solutions to reduce the burden on operating room personnel [14], the literature also reports insufficient or incomplete separation of airborne particles [8,26].

Operating rooms in hospitals are generally equipped with mechanical ventilation according to national regulations and applicable hygiene requirements (e.g., in Germany, DIN 1946-4). This ventilation reduces the number of airborne germs and particles and evacuates the heat generated, as well as any hazardous substances. Nevertheless, general air conditioning systems or laminar air flow ceilings are not sufficient to capture surgical smoke near the source. Instead, they spread it into the operating room, causing olfactory discomfort.

### 1.4. Standards and Recommendations for Prevention of Surgical Smoke

In the USA, the use of local exhaust ventilation (LEV) systems has been recommended by professional associations of perioperative nurses [27,28,29] and government agencies [30,31]. Comparable standards are published in the United Kingdom [32,33]. In Germany, professional associations in the surgical and nursing field have not yet dealt significantly with the topic. There are only the following two papers available, both of which were published by health and safety initiatives:

1. Recommendations of a consensus working group of the International Social Security Association (ISSA) based on risk assessments of activities involving exposure to surgical smoke [34];
2. Chapter 8.1, “Surgical smoke” was added to the Technical Rules for Hazardous Substances (*Technische Regel für Gefahrstoffe*, German abbreviation TRGS), no. 525. This version was implemented in German occupational health and safety legislation in September 2014 [35].

In summary, the following is recommended:

- Lasers, electrosurgical units, and other medical devices involving intense smoke production should only be used in work areas equipped with a ventilation system (filtration at least with high-efficiency particulate air filter);
- Smoke should, as far as possible, be captured at the source;
- In poorly ventilated areas it is necessary to use portable smoke evacuators equipped with active charcoal filters;
- Special individual protective measures (eye protection and high-filtration masks) during surgical procedures are regarded as unnecessary when smoke evacuation systems are adequate. In case of nonappropriate protection against the particulate components of surgical smoke, particle filtering half masks (class FFP2) or higher are recommended, even if they do not protect against the nanometric fraction of these particulate components;
- Furthermore, all workers should be trained and informed of the hazards of surgical smoke and of preventive measures according to the Occupational Health and Safety Framework Directive 89/391/EEC [36];
- Given current scientific knowledge, no specific preventive medical surveillance of personnel exposed to surgical smoke is necessary. However, occupational health surveillance medical records should include components that allow exposure to be traced.

### 1.5. Hazard Awareness of Operating Room Staff and Application of Protective Measures in Practice

Authors of previous and recent surveys among operating room nurses and surgeons unanimously concluded that the use of protective measures is not widespread. This applies both to the existence of such measures and to the adherence of their use in the event of exposure [37,38,39,40,41]. Surgeons, although they do not feel sufficiently informed about the actual health risks [40], often do not use smoke extractors and consider surgical diathermy to be a safe procedure [42]. Hill et al. [5] stated, in a UK study, that smoke extraction systems were only available in 66% of 50 plastic surgery units investigated.

As Ball [39,43] reported, perioperative nurses regarded the lack of equipment and the noise of the technical system, as well as staff compliance and physician resistance, as obstacles to adherence to smoke evacuation recommendations. Resistance obviously occurs frequently, especially in electrosurgery [41]. Surgeons’ resistance or refusal to allow the use of local exhaust ventilation (LEV) was also reported by Edwards and Reiman [36] as the most common obstacle to the provision of protection measures.

The extensive study by Steege et al. [41] with over 4500 respondents provides a good overview of various protective measures in operating rooms in the USA. Only half of the respondents reported that LEV was always used during laser surgery and a minority of the respondents (14%) said this was true during electrosurgery. Most respondents reported never wearing any special respiratory mask and 74/39% of the staff exposed to surgical smoke during laser and electrosurgery wear special eye protection. Furthermore, 49% of laser surgery and 44% of electrosurgery respondents said that they had never been received training about the hazards of surgical smoke, and another third of the respondents were trained more than 12 months ago.

In addition, Chapman et al. [44] found that of 153 dermatology residents in hospitals, less than half of them knew there was a smoke evacuation system available and 88% said they did not wear high filtration masks. The authors pointed out that these were more expensive than normal surgical masks. Despite the low reported use of protective equipment, 72% of respondents were concerned about carcinogens in surgical smoke and 73% of respondents felt that adequate precautions were not being taken to protect them from surgical smoke. The same number never received education on the hazards of electrosurgery smoke. Tregoning, Bigony and Lewin et al. [12,45,46] also stated that resistance to smoke evacuation can be attributed to a general lack of knowledge, but also to expense, inconvenience, and time constraints.

According to Marsh [47], in addition to lack of knowledge about the risks of surgical smoke and the insufficient protective power of surgical masks, the cost of a smoke evacuation system and the significant price difference between a standard face mask and a high filtration face mask can be regarded as obstacles for the implementation of effective smoke evacuation procedures and preventive measures.

In the survey by Edwards and Reiman [38], 72% to 76% of 1356 respondents answered that a wall suction system was “always/often used during electrosurgery, depending on the procedure, and 54% to 82% said it was used during laser surgical procedures. Portable smoke evacuators during electrosurgery were said to be used by between 12% and 24% (with the exception of condyloma or dysplasia treatment, 68%) and similarly between 1% and 21% during laser surgery.

To gain insight into the situation of equipment and application adherence, we conducted a mixed method study consisting of (A) a standardized survey of surgeons and operating room nurses and (B) semi-standardized telephone interviews with manufacturers and distributors of suction systems for surgical smoke in operating rooms. Our research questions in part A were:

- To what extent is surgical smoke perceived as a health hazard? What do operating room personnel know about health hazards and protective measures?
- Which protective measures are in place in operating rooms in hospitals and outpatient facilities at a structural and behavioral level of prevention?
- To which extent are existing prevention measures implemented in practice? What do employees want from employers/colleagues and from producers of technical devices regarding protection from surgical smoke?

In addition, we were interested in systematic differences between the answers of respondents in hospitals and outpatient facilities, as well as those between surgeons and operating room nurses.

Analogously to part A, we wanted to specify, from the perspective of the interview partners in part B, the following:

- can users, in general, be regarded as sufficiently informed;
- does an own market assessment indicates an increasing demand for suction systems in recent years;
- do surgeons use the existing devices; and
- do situations differ at hospitals and outpatient facilities.

## 2. Materials and Methods

### 2.1. Part A: Survey

#### 2.1.1. Recruitment

The survey was conducted online with a self-developed questionnaire following pretests between October and December 2018. Incomplete datasets were mostly avoided through completion control which was implemented in the online tool.

Access to surgeons took place with the support of a commercial address provider. Surgeons were invited by email and the invitation was followed by a reminder after four weeks. Addressed medical specialties were general, vascular, visceral, cardiac, pediatric, oral/maxillofacial, neuro, orthopedics/trauma, plastic, and thoracic surgery, as well as the disciplines of dermatology, urology, gynecology and otorhinolaryngology (3620 were senior hospital physicians and 3875 in outpatient facilities).

Operating room nurses were recruited with the support of the two professional associations of surgical technologists and anesthesiology assistants, which forwarded the invitation to the investigation to their members (full recruitment, data base *n* = 150 and *n* = 69).

#### 2.1.2. Survey Instrument

Besides information on sociodemographic and professional characteristics, survey questions covered the following:

- perceptions about the health hazards of surgical smoke (own and in the working team) in the department in which most of the working time was spent;
- the state of respective knowledge and behavior and the existence of technical prevention measures and personal protective equipment, (PPE) at the workplace, as well as perceptions of changes in preventive behavior at the workplace within the last four years (since the beginning of 2015);
- the use of preventive equipment, if available;
- barriers (reasons if equipment was not “always” used; standardized specification of 5 to 10 multiple answers);
- wishes and suggestions for improvement, addressed to employer, colleagues, and employees, as well as to manufacturers of technical devices.

Parts of the instrument were derived from the questionnaire by Steege et al. [41].

#### 2.1.3. Statistical Analysis

Results are described by percentage and mean/standard deviation/median. To identify differences between hospitals and outpatient facilities and between surgeons and operating room nurses, bivariate Chi^2^ tests with a significance threshold of *p* ≤ 0.05 were carried out. The respective effect sizes (contingency coefficient C (*CC*) and *phi*) were classified as <0.3, <0.5, and ≥0.5, indicating a low, moderate, or high effect size. The CC was used also in the case of presenting dichotomized results. For better legibility, percentages are also presented for fewer than 100 cases.

### 2.2. Part B: Interviews with Manufacturers of Smoke Suction Devices and Distribution Companies

In total, 12 enterprises which produce suction systems for surgical smoke and 11 distribution companies were contacted. Key questions for interviews with company representatives with customer experience were:

1. Has there been an obviously increasing demand for suction systems in recent years? Do surgeons use existing devices? If not: For what reasons? Are they aware of health hazards?
2. Are users sufficiently informed about prevention measures to avoid surgical smoke? What role do official prevention recommendations play in practice?
3. Which differences can be observed between the situation and behavior in hospitals versus outpatient facilities?

## 3. Results

### 3.1. Part A: Survey

#### 3.1.1. Response Rate and Sample Description

After exclusion of *n* = 47 and 359 random sample neutral failures (1% and 9% of hospital surgeons and outpatient surgeons, respectively), addressable target groups of *n* = 3573 and *n* = 3516 could be defined. The overall response rate was 6.5% (8.6% hospital surgeons, 1.5% outpatient surgeons, and 64.8% operating room nurses). A total of *N* = 359 surgeons were analyzable (*n* = 306 in hospitals, *n* = 53 in outpatient facilities) and *n* = 142 operating room nurses (*n* = 140 in hospitals).

The median occupational exposure to surgical smoke was 23 years for surgeons and 10 years for operating room nurses. The median number of operations using laser or electrical technology in the last full working week was 10 (surgeons) and 19 (operating room nurses). Ninety-one percent of respondents in an outpatient setting were practice owners or respective partners. In hospitals, 59.3% of the surgeons indicated one professional specialization (*n* = 213); others, more than one (maximum 10, *n* = 146). More sample characteristics can be derived from Table 1.

#### 3.1.2. Perceptions: Is Surgical Smoke a Health Hazard?

About half of the surgeons rated health hazards of surgical smoke without protective measures as “high/very high” and had already thought about it “frequently/very often” (see Table 2). Operating room nurses were much more concerned about health hazards than surgeons. Only a few surgeons and assistance personnel felt properly informed about possible health hazards. However, if only surgeons are considered, the level of information in the outpatient sector is negligibly higher than in hospitals (32.1% vs. 13.7%, *p* = 0.000, and *CC* = 0.22). When they thought about their own working team, 65.4% of respondents in outpatient facilities, and significantly more in hospitals (86.1%), thought that awareness of health hazards in the team was “low” or “non-existent”.

Concerning the whole working area, 15.5% of hospital respondents and 23.6% from outpatient facilities saw increased awareness of the health hazards posed by surgical smoke within the last four years (since the implementation of the amendment of the TRGS 525); others assessed the awareness as “non-existent” or “the same”. The perception of surgeons in outpatient facilities was slightly better but could not be confirmed statistically (*p* = 0.065 and *CC* = 0.12).

#### 3.1.3. Availability and Use of Protective Measures in the Workplace

Six technical and individual measures were specified and questioned with regard to the operating room in which respondents spent most of their working hours as follows: (1) Air conditioning system, (2) laminar air flow ceiling above the surgical site, (3) stationary suction system, (4) portable suction system, (5) personal protective equipment (PPE) including special eye protection (not own glasses), and (6) special protective mask (not standard surgical mask). The data has been classified into smoke suction systems and air condition or laminar air flow systems without special smoke extraction systems, each with and without PPE. Additionally, the existence of work plans for protection against smoke exposure (e.g., how to keep distance from the risk source) and of information on health hazards was surveyed.

As can be derived from Table 3, in total, 52.1% of respondents in hospitals and a few more in outpatient facilities (65.5%) reported the existence of any type of special suction system to capture surgical smoke. Nearly half of the respondents in hospitals thought that surgical smoke could at least be captured by air conditioning or a laminar air flow system (43.5% + 3.2% including the availability of PPE).

In detail, technical systems and personal protective equipment in hospitals are available as follows: 46.0% smoke evacuators, 12.3% stationary evacuation systems, 60.1% laminar air flow ceilings, 74.5% air conditioning systems, 12.6% special protective masks, and 24.9% special eye protection. The respective percentages in outpatient facilities were 61.8%, 10.9%, 18.2%, 49.1%, 18.2%, and 36.4% (no table).

Prevention work plans and available information about health hazards were reported in half of the hospital operating rooms and little more from outpatient facilities (see Table 3).

One-fifth of respondents from hospitals reported that technical measures had improved over the past four years, and 10% said that the availability of personal equipment had improved. The results in outpatient facilities are comparable. However, access to information on health hazards has improved in outpatient facilities by about a quarter.

Nearly half of the surgeons in hospitals, and significantly fewer outpatient surgeons, had no experience with instructions in technical or smoke-avoidant working techniques (see Table 3). The same was true for 22.9% of operating room nurses regarding protective equipment in hospitals (no results for those in outpatient facilities due to the low number of *n* = 2). Hospital surgeons and operating room nurses differed slightly concerning their experience (52.6% vs. 77.4%, respectively, with at least one instruction session, *phi* = 0.23 and *p* = 0.000).

Of those with experience, 34.3% of surgeons in hospitals, 25.7% of surgeons in outpatient facilities, and 37.6% of hospital operating room nurses reported that the last instruction they received regarding technical equipment occurred more than two years ago (*n* = 140/35/140, respectively). Analogously, 43.8%, 35.3% and 38.8% reported this to be the case for personal protective equipment (*n* = 96, 34, and 137), and 44.6%, 36.4%, and 36.5% for smoke-avoidant working techniques (*n* = 74, 22, and 138).

Surgeons who owned practices in outpatient facilities were asked how familiar they were with the two above-mentioned published recommendations. The question was only addressed to them because, as employers, they are responsible for occupational health and safety according to German occupational health and safety law (*n* = 49 out of 55).

In total, 61.2% (*n* = 30) were familiar with TRGS 525, although it had been read by only half of them. The ISSA guideline, which is several years older, was known to 30.6% (*n* = 15), and had been read by *n* = 4. When asked for the basis of their decision regarding technical protective measures, 67.3% (*n* = 33) named the recommendations of experts or colleagues and 51.0% (*n* = 25) cited sector-related written informational material. Exposure measurements had been carried out in only one facility.

#### 3.1.4. Obstacles to the Use of Preventive Measures in Practice, and Requests

The global question to surgeons regarding the extent to which they pay attention to avoiding surgical smoke during surgery was answered with “mostly” or “always” by 50.8% of hospital and 69.8% of outpatient facility respondents (see Table 4). For hospital surgeons and outpatient facility surgeons, respectively, the most important reasons given in free text for “rarely” or “never” considering avoidance, albeit with small percentage differences, were a lack of problem awareness or thoughtlessness (20.0% and 25.0%); fatalism, since smoke is part of professional activities (20.0% and 6.3%); concentration on the procedure or patient safety (14.7% and 18% to 8%); time pressure (12.0% and 12.5%); and the perception of a low health risk. Furthermore, with percentages of less than 10%, surgeons stated that they were not or badly informed; that there was no or inadequate technology available; that the procedure time was too short/the smoke development too low; that there was ignorance, lack of interest, idleness, or habits; that devices were too unwieldy or protection too complex; and that there were no standard operation procedures or inexperienced personnel.

In addition, all those surveyed were asked how frequently technical smoke extraction systems or special personal protective equipment were used, if available, during the last full working week with smoke exposure. In hospitals, if available, portable smoke evacuators were “mostly” or “always” used by 45.0% (see Table 4).

When asked about (standardized operationalized) reasons for mostly non-use (“sometimes” or “never”), 17.1% reported that another system was used for smoke capture. Often, namely 28.8% of respondents, said that the device would be forgotten and no one would care about it. Other more frequently mentioned reasons were very low smoke emission (26.1%) and the loud noise of the device (22.5%). In addition, 17% to 6% (in this order), mentioned a sufficient general air conditioning system, the bulkiness/complexity of the device, its non-availability due to use in another work area, or a prohibition by the surgeon. For outpatient facilities, no valid information is interpretable due to the lack of cases. However, results can be derived from Table 4.

When asked about their requests to employers, colleagues, and employees, 45.6% of surgeons listed one or more items concerning more or better information about health hazards (free text multiple answers, categorized into single-answer types). Furthermore, 35.7% wanted more or better protective measures (evacuation systems, PPE at least one answer) and 18.7% more occupational safety culture or awareness of hazards and acceptance of risks (database, *n* = 299 responses from 241 respondents).

Operating room nurses responded similarly but differed slightly from surgeons in the number of verbally expressed needs (42.4%, 34.7%, and 22.9%; *r* = 0.11 and *p* = 0.010; database, *n* = 299 responses from 241 respondents).

Furthermore, both occupational groups were asked about their requests to manufacturers of extraction systems and *N* = 235 responded with a total of 280 answers. From a technical point of view, the following were desired: 14.9% quieter smoke evacuators; 14.5% easier handling; 13.2% better functioning (global); 11.5% standard suction device, integrated in the surgical instrument; 3.4% more comfortable protective masks; 3.0% smaller and lighter portable devices; 0.4% better device filter performance; and 0.4% better compatibility with the technology of other manufacturers.

Nontechnical requests were for more or better instructions for using the devices (26.0%); more or better information about health risks and the benefits of prevention (19.1%); less expensive devices or protective masks (7.2%); more forceful marketing (e.g., free samples, more offers, more presence in the facility), and more “pressure” on employers to invest in prevention (5.5%).

### 3.2. Part B: Interviews with Manufacturers and Distributors of Smoke Suction Devices

In total, semi-standardized telephone interviews with representatives of five out of 12 enterprises which produce suction systems for surgical smoke and three out of 11 distribution companies were contacted (eight interviews). The answers to the key questions (Increasing demands for suction systems in recent years? Use of existing devices and reasons for not using? Awareness and knowledge of health hazards? and Differences between hospitals and outpatient facilities?) are summarized as follows:

- The demand for technical protective measures was described by all respondents as restrained and lower than in other countries, e.g., the USA (six responses of *n* = 5 persons);
- A slight overall upward trend was observed regarding market changes for technical devices in recent years (since the amendment of the TRGS at the beginning of 2015). However, sudden changes, e.g., due to the publication of a new technical rule could not be observed (five responses of *n* = 5);
- Five respondents said that, according to their experience, purchased equipment seems to be used and one person emphasized the importance of a “caretaker.” However, one manufacturer concluded that from the mismatch between the number of pieces of equipment purchased and the reordering of filters, consequently, use of the equipment was unlikely. Another knew this was the case from his own experience (seven answers of *n* = 7);
- All interviewees made clear that the topic was of little importance in practice; one interviewee referred to the significantly higher awareness of users, for example in the USA (four answers of *n* = 4);
- The level of knowledge of health hazards and prevention options was unanimously regarded as low. Two interviewees referred to the practical irrelevance of existing standards as a possible reason for this (seven answers of *n* = 7).

When asked about possible obstacles to the non-use of protective measures from their own perspective, respondents gave answers which were classified into the following categories:

1. **Lack of knowledge (seven answers of *n* = 5 persons)** Lack of knowledge is also seen as an important barrier to the appropriate use of protective measures. Many customers of the interviewees think that surgical smoke “disappears” via normal room ventilation or that the laminar air flow ceiling system or a surgical mask is at least partially a sufficient protective measure. However, two respondents emphasized that many physicians are generally aware of the hazards of surgical smoke, but do not put this knowledge into practice or are “resistant to advice,” which can also be due to financial considerations.
2. **Hierarchical relationships in the operating room (six answers of n = 3**) At the interpersonal level, the lack of influence that operating room nurses have on the promotion of prevention issues in Germany was repeatedly deplored. It was emphasized that occupational health and safety is more important in other countries due to the influence of this occupational group.
3. **Lack of practicability of suction devices (four answers of *n* = 2)** The experts were aware of the unwieldiness and weight of portable suction devices; however, they were also hopeful that the devices would be better accepted due to recent technical innovations regarding their weight.
4. **High noise level of devices (three answers of *n* = 3)** The volume of devices is also noted as a usage obstacle. One interviewee pointed out that devices are often used with maximum extraction power, even during minor interventions. This keeps the noise level unnecessarily high where lower extraction power would be sufficient.
5. **Lack of legal obligation to use suction equipment (six answers of *n* = 6)** Half of the interviewees voted for legal obligations for the appropriate use of suction equipment in order to overcome low awareness of the subject.
6. **High purchase and maintenance costs of extraction devices (eight answers of *n* = 6)** The high cost of extraction devices is seen as a very important reason for not taking preventive measures. However, it seemed inconceivable to one interviewee that investment costs for small subsystems are a major problem.
7. **Differences between hospitals and outpatient practices (six answers of *n* = 5)** The majority of respondents emphasized a lower demand for suction devices in outpatient practices. They found this fact surprising, as the odor nuisance is much higher than in hospitals due to smaller rooms and fewer air conditioning systems. One interviewee confirmed that hospital staff had a higher level of information regarding official recommendations for prevention.

## 4. Discussion

This study was the first to systematically evaluate the state of surgical smoke prevention in Germany. In addition, we gained insight into the state of knowledge and the corresponding attitudes of staff in operating rooms, both from the point of view of surgeons and technical assistants in the inpatient and outpatient sector.

With regards to the existence of technical prevention, almost all facilities had protective measures. These included air conditioning and laminar air flow ceiling systems, which, however, are often insufficient for the complete evacuation of surgical smoke. Only half of hospital respondents and almost two-thirds of those from outpatient facilities reported using special suction devices. Stationary extraction systems were particularly underrepresented. Although general vacuum suction systems are in place in hospital operating rooms, their use with special suction nozzles for smoke evacuation is rather seldom. It is possible that respondents only thought of “special systems” when answering the respective question. Therefore, we do not consider the results regarding the existence of stationary suction systems to be valid but see them rather as an expression of a low culture of prevention in that field.

Consistent with other studies mentioned above, personal protective equipment, namely eye protection and high-filtration particle masks, obviously plays a minor role. Both measures are recommended if a certain exposure is exceeded but are not necessary when technical measures are in use [1]. However, one limiting factor of our study design is that we cannot consider the link between real exposure and the appropriate use of equipment in detail.

Another characteristic of “expandable” occupational health and safety culture is the low level of experience with instruction in technical equipment, PSE, or smoke-avoidant working techniques.

Inclusion should generally be given higher priority, as confirmed by another German study; a quarter of the operating room nurses in the German “Operating Room Barometer” who were asked about their work situation said they were insufficiently trained in the handling of new equipment [48].

Since the amendment of the TRGS 525, only one-fifth to one-quarter of the respondents noticed general improvements in technical protections against surgical smoke. These rates are compatible with evaluations in other areas of self-protection at work, for example, pest control and bed disinfection, according to internal surveys of the German Statutory Accident Insurance and Prevention in the Health and Welfare Services (BGW). The problem of adherence to occupational self-protection appears to be fundamental. It is also demonstrated in examples dealing with other health risks [49,50].

The published recommendations are not satisfactorily known, as shown by the answers of owners of outpatient practices. Our industrial interview partners also recognized the lack of awareness and complained about the recommendations’ limited practical suitability due to language that is “too scientific” and “not target-group oriented”.

In addition, our interviewees also recognized a slight general improvement in the prevention situation in recent years, although they could not directly associate it with the introduction of official recommendations.

As far as the awareness of surgical smoke as a potential health hazard is concerned, we consider that the proportion of half of the surveyed surgeons to be low. This could be true also in view of the low level of detailed information on actual exposure per operation procedure in this study. The assessments from our interview partners were mixed in this respect, although surgeons are generally considered reluctant in terms of their problem awareness and willingness to invest.

In fact, operating room nurses are significantly more concerned about possible health risks, although this occupational group (see e.g., the study by Ball [39]) does not always consistently follow smoke evacuation recommendations. It is regrettable that surgical assistants in Germany have comparatively little influence in the field of prevention due to the culture of professional hierarchy. We also see nothing on the topic in profession-related journals. This is different from, for example, the USA, where perioperative nurses are very present in scientific publications (see e.g., [27,39,46,51,52]). However, physicians’ resistance to their own health protection seems to be frequent [39,41,42], which also plays a certain role in the prevention obstacles mentioned in our study.

Consequently, the inadequate use of existing protective measures in practice coincides with other evaluations [37,38,39,40,41]. More consistent instructions and updates in the use of technical equipment and smoke-avoidant working techniques would hopefully improve deficits and might counteract the fatalism and lack of problem awareness of surgeons.

The most frequently cited reason for a lack of use of portable suction devices in hospitals was that it was not part of the standard working procedure (SOP). As a result, a change in SOPs should be recommended which formalizes and explicitly focuses on occupational health and safety.

The statistically better situation in the few outpatient practices that responded as compared with hospitals must be viewed critically. The differences are mostly small and should not be overestimated. Even if the survey results coincide with the assessments of manufacturers and distribution companies for the most part, interviewees could not discern a better prevention situation in outpatient practices. However, the higher level of knowledge of the outpatient practice owners in our study is likely due to their responsibility for occupational safety.

## 5. Limitations

Even if there is not a defined “sufficient” response rate for electronic surveys among surgeons [53], the very low values in our study (5% of surgeons and, especially, in outpatient facilities 2%) definitely implies a general overestimation of positive findings and strongly limits the representativeness of the results. In comparable studies, response rates differed between 14% [38] and 95% [40]. The use of a paper questionnaire for surgeons would possibly increase the response rate [54].

In addition, the difference between self-assessment and assessment of others (team) concerning awareness of health hazards is an indication of a positive selection of respondents. The most important reason for the low response rate among surgeons might be their aforementioned lack of interest combined with a well-known lack of time.

Further limitations are the retrospective study design with exclusively subjective assessments. A pre-post comparison before and after the amendment of TRGS 525 would have been more advantageous. Moreover, we have no information about perceptions, attitudes, and experiences of assistant staff in outpatient practices, which are not organized in the two cooperating professional associations. Finally, the study does not provide more detailed insights into prevention behavior depending on detailed surgical procedures or the duration of exposure to surgical smoke. The same is true for a lacking distinction between the perception of chemical and biological risks.

## 6. Conclusions

Regularly updated hazard assessments for operational exposure are indispensable. Close cooperation should be established between occupational safety specialists and occupational health physicians. The implementation of OH&S recommendations in Standard Operating Procedures (SOP) is also recommended.

Surgeons and assistant staff should be better informed about the possible health risks of surgical smoke and its prevention. Awareness rising can take place through regularly updated instruction and other formalized training such as a facility-based initiative [42] and manufacturers of smoke evacuation devices should be invited to support regular onsite training [55].

Operation room nurses can play a more active role as moderators and advocates for their own health and that of their team by collecting knowledge and implementing preventive processes. Successful results can be derived from other countries [39,43,46,51,56].

In some places, this requires a rethinking of the interprofessional distribution of tasks. An American intervention study showed that if assistance staff is actively involved in risk assessment processes, personnel receive appropriate training and, in addition, an audit system for monitoring equipment usage is implemented and occupational health and safety can be protected [27,57]. A key component of the award-winning program was an interdisciplinary implementation team with facility coordinators who performed a gap analysis, coordinated between departments, identified champions, developed communication plans to address identified concerns, encouraged team involvement, and educated staff members about smoke-free working procedures.

A positive attitude on the part of the management is demonstrably an important supporting factor in achieving prevention goals [39,43,51,55,56]. Moreover, surgeons with positive attitudes should be identified as stakeholders and role models [13,38].

Legal mandating of existing recommendations, as requested by manufacturers and distributors, is not possible according to the current circumstances. First, further studies with resilient dose-response exposures to health hazards are needed [18]. In order to make existing recommendations better known, more intensive general information strategies based on easy-to-understand brochures are recommended.

Regarding healthcare research, long-term follow-ups for the development of preventive behavior are necessary to identify early obstacles. Accordingly, better support should be available for facilities and staff, for example, from German statutory accident insurance consultants and labor protection authorities.

Lastly, since the cost of suction devices is an important argument against their implementation by actors in the ambulant setting, a cost-benefit analysis of some of the more popular devices could be of particular interest [18].

## Figures and Tables

**Table 1:** Sample characteristics.

Personal Characteristics ^(1)^	Surgeons (*n* = 359)	Operating Room Nurses (*n* = 142)
Total professional exposure to surgical smoke (years; *n* = 359/142) ^(2^^)^	23.0 (7.8), MED = 23	9.8 (7.3), MED = 7
Total work experience (years, current profession; *n* = 358/142)	22.7 (7.8), MED = 22	9.8 (7.4), MED = 7
Age (years; *n* = 358/142) ^(^^2)^	51.1 (4.7), MED = 51	32.0 (9.4), MED = 29
Sex (female; *n* = 359/142)	18.9% (*n* = 68)	81.0% (*n* = 115)
Surgical specialties (proportion of multiple responses referring to “classical” specialties (532 answers, 359 persons/315 answers, 142 persons) ^(^^3)^	67.5% (*n* = 532)	29.8 (*n* = 315)
Professional status (medical staff)		
Hospital: Medical director or senior physician (*n* = 305 of 306) ^(4)^	99.7% (*n* = 304)	---
Outpatient facility: Owner (*n* = 53 of 55)	96.2% (*n* = 51)	---
Exposure to surgical smoke (*n* = 354/141) ^(5) (6)^		
Number of procedures with use of laser or electrosurgery during the last complete working week ^(2)^	15.6 (34.3), MED = 10	19.2 (14.3), MED = 18
Total number of these surgery hours:		
1–5 h	45.8% (*n* = 161)	58.2% (*n* = 82)
6–15 h	27.1% (*n* = 96)	12.1% (*n* = 17)
≥16 h	27.1% (*n* = 96)	29.8% (*n* = 42)
**Facility characteristics**		**Percent (n)**
Hospital type (*n* = 446):	Maximum care	44.4% (*n* = 198)
	Standard care	40.6% (*n* = 181)
	Basic care	12.6% (*n* = 56)
	Specialized hospital	2.5% (*n* = 11)
Number of employees (*n* = 446)	1–99	12.8% (*n* = 57)
	100–499	29.4% (*n* = 131)
	≥500	57.8% (*n* = 258)
Type of surgical methods in the current work area (*n* = 446)	Electrosurgery	99.8% (*n* = 445)
	Laser surgery	24.7% (*n* = 110)
Outpatient facility: Type (*n* = 55)	Practice/Surg. center	87.3 (*n* = 48)
	Practice clinic	5.5 (*n* = 3)
	Med. care center	7.3 (*n* = 4)
Outpatient facility: Number of employees (*n* = 55)	≤10	69.1 (*n* = 38)
	11–99	25.5 (*n* = 14)
	≥100	5.5 (*n* = 3)
Outpatient facility: Type of methods in the current work area (*n* = 55)	Electrosurgery	96.4 (*n* = 53)
	Laser surgery	50.9 (*n* = 28)

^(1)^ Number of valid databases in brackets (surgeons and operating room nurses); ^(2)^ mean value (standard deviation), median (MED); ^(3)^ according to the list of “German Medical Specialties” (Model Regulations on Continuing Medical Education, March 2008). Most frequent specializations (532 multiple answers of surgeons, general surgery (18.4%), visceral surgery (15.2%), trauma surgery and traumatology (14.1%), orthopedics (13.3%); 315 multiple answers of operating room nurses, trauma surgery and traumatology (12.7%) and general surgery (9.5%); ^(4)^ proportion of medical directors (8.5%); proportion of missing values in this group (15.3%); ^(5)^ exposure in the last full working week in the area where most time was spent; ^(6)^ missing data, 5 surgeons and 1 operating room nurse not involved in laser or electrosurgery in the last four weeks.

**Table 2:** Awareness of health hazards caused by surgical smoke.

**Own Awareness of (*n* = 446/55)**	**Surgeons (%)**	**Operating Room Nurses (%)**	**Effect Size/Significance**
Health hazards without protective measures assessed as high/very high (vs. somewhat low/not present)	50.4	88.0	*CC* = 0.36; *p* = 0.000
Frequently/very frequently thought about health hazards (vs. somewhat rarely/never)	44.6	70.4	*Phi* = 0.36; *p* = 0.000
Well informed about possible health hazards (vs. not well informed)	16.5	15.5	n.s.
**Awareness in the Work Team (*n* = 446/55)**	**Hospitals (%)**	**Outpatient Facilities (%)**	**Effect Size/Significance**
Awareness of health hazards in the team low/not available (vs. very/relatively good)	86.1	65.4	*CC* = 0.33; *p* = 0.000
Health hazards already a topic in the work area/team (vs. no)	55.6	52.7	n.s.
Increased awareness of health threats across the work sector over the last four years (since 2015; vs. stable/depressed)	15.5	23.6	*CC* = 0.12; *p* = 0.065

Abbreviations: *CC*, contingency coefficient effect size for >2 × 2 fields Chi^2^ test; n.s., not significant; *phi*, effect size for 2 × 2 fields Chi^2^ test.

**Table 3:** Existence of technical, personal, and organizational protection measures and instructions regarding smoke avoidance procedures.

**Technical/Personal Protection Equipment (*n* = 446/55) ^(1)^**	**Hospitals (%)**	**Outpatient Facilities (%)**	**Effect Size/Significance**
Smoke suction system ^(2)^ + PPE	11.2	18.2	*CC* = 0.24; *p* =0.000
Smoke suction system without PPE	40.8	47.3	
Air condition or laminar air flow system (no LEV) + PPE	1.3	0.0	
Air condition or laminar air flow system (no LEV) without PPE	43.5	18.2	
None (no answer)	3.2	16.3	
**Existence of Organizational** **Protection** **Measures** **(*n* = 445/55)**	**Hospitals (%)**	**Outpatient Facilities** **(%)**	**Effect Size/Significance**
Work plans to protect against surgical smoke (e.g., keeping distance from the source of risk)	51.8	69.1	*Phi* = 0.11; *p* =0.015
Information on health hazards	51.2	78.2	*Phi* = 0.17; *p* =0.000
**Improved Protection Measures over the Last Four Years ^(3)^**			
Technical measures (*n* = 444/55)	20.5	25.5	−
Special PPE (*n* = 438/53)	10.0	17.0	−
Work plans (*n* = 444/55)	3.8	16.4	*CC* = 0.19; *p* = 0.000
Information on health hazards (*n* = 443/55)	6.1	23.6	*CC* = 0.23; *p* = 0.000
**Experience with Instruction in Smoke Avoidance Measures (vs. None) ^(4)^**	**Hospitals (%)**	**Outpatient Facilities (%)**	**Effect Size/Significance**
Surgeons (*n* = 304/52)	52.6	77.4	*Phi* = 0.18; *p* = 0.001
Operating room nurses (hospitals; *n* = 140)	−	77.1	−

Abbreviations: *LEV*, local exhaust ventilation; *PPE*, personal protection equipment; also see Table 2. ^1^, Sorted according to protection quality against health hazards of surgical smoke; ^2^, stationary or portable systems; ^3^, since amendment of TRGS 525, Chapter 8.1 “Surgical smoke—hazards and protective measures” 2015; and ^4^, technical devices for the capture of surgical smoke, special PPE or smoke-avoidant working techniques.

**Table 4:** Use of protective measures in practice.

Existence of Organizational Protection Measures (Total Data Base: *n* = 445/55 in Hospitals/Outpatient Facilities)	Hospitals (%)	Outpatient Facilities (%)	Effect Size/Significance
Surgeons: Attention to the avoidance of surgical smoke when operating “most/ever” (vs. sometimes/never; *n* = 305/53)	50.8	69.8	*Phi* = 0.17; *p* = 0.014
**Use of existing smoke suction systems “always/mostly” (vs. sometimes/never)**			
Stationary suction system (*n* = 55/6)	32.7	66.7	(---)
Portable smoke evacuator (*n* = 202/34)	45.0	85.3	*CC* = 0.17; *p* = 0.014
**Use of existing personal protective equipment “always/mostly” (vs. sometimes/never)**			
Special eye protection (*n* = 109/20)	24.8	36.4	*CC* = 0.28; *p* = 0.028
Special protective mask (*n* = 55/10) ^(1)^	12.0	18.9	n.s.

Abbreviations: (---), no tests due to low case number and ^1^, powered air purifying respirator with filter, breathing protection mask with or without activated carbon filter.
